# Transforming European Food Systems with multi-actor networks and Living Labs through the FoodSHIFT Approach

**DOI:** 10.12688/openreseurope.17982.2

**Published:** 2024-10-25

**Authors:** Luke John Schafer, Aida Anthouli, Alessandra Schmidt, Anita Beblek, Annika Fruehbeisser, Beatrice Walthall, Carla Mingolla, Catalina Rogozan, Damiano Petruzzella, Dirk Wascher, Francesca Volpe, Giordano Ruggeri, Gustavo Arciniegas, Jose Luis Vicente-Vicente, Katerina Riviou, Katerina Valta, Lena Marijke Wenzel, Lorenzo Labellarte, Maarten Crivits, Malgorzata Swiader, Marin Lysak, Marta Sylla, Poppy Eyre, Raluca Barbu, Stefano Corsi, Christian Bugge Henriksen

**Affiliations:** 1 Department of Plant and Environmental Sciences, University of Copenhagen, Copenhagen, Capital Region of Denmark, 2630, Denmark; 2 DRAXIS Environmental S.A, Athens, Greece; 3 Fab Lab Barcelona, Institute for Advanced Architecture of Catalonia, Barcelona, Catalonia, Spain; 4 agrathaer, Berlin, Germany; 5 (ZALF), Leibniz Center for Agricultural Landscape Research, Muncheberg, Germany; 6 ILVO, 2. Eigen Vermogen Van Het Instituut Voor Landbouwen Visserijonderzoek, Flanders, Belgium; 7 Highclere Consulting SRL, Brasov, Romania; 8 CIHEAM Bari, Bari, Italy; 9 Susmetro - Sustainable Design for Metropolitan Landscapes, Tilburg, Netherlands Antilles; 10 Department of Agricultural and Environmental Sciences, University of Milan, Milan, Italy; 11 Ellinogermaniki Agogi, Athens, Greece; 12 Institute of Spatial Management, Wrocław University of Environmental and Life Sciences, Wroclaw, Poland

**Keywords:** Food System Living Lab (FSLL), Sustainable Transformation, Multi-actor network, Place-based, Citizen-driven, Systems-thinking

## Abstract

Our current global Food System is facing extraordinary challenges in both size and severity, including a rise in unsustainable consumption behaviours, continued environmental degradation, growing food insecurity, and widening social inequalities. A Food System transformation is now both critically important and overwhelmingly complex, requiring nothing less than a complete overhaul of the entire value chain. Everyone is needed: Small Medium Enterprises (SMEs) with technological solutions, Non-Governmental Organisations (NGOs) with social innovations, researchers with novel methodologies, governments with food policy advancements, professionals with varying expertise, and last but not least, empowered and informed citizens with the ability and resources for better decision-making. Living Labs offer a holistic, place-based approach needed to facilitate multi-actor inputs on various levels, specifically Food System Living Labs (FSLLs) like the ones established as part of the FoodSHIFT 2030 Project. Nine front-runner Food System Living Labs were operationalised alongside a novel framework merging high-level interdisciplinary initiatives with a diverse set of innovative approaches towards more Sustainable Food Systems (SFS). The FoodSHIFT Approach concept was praised by external evaluators for its ground-breaking framework, and the nearly completed project has been listed as a best practice. However, positive applications alone will not ensure a cross-sector European-wide Food System transformation, and the following text offers a critical reflection coupled with experience-based solutions to further improve the FoodSHIFT Approach.

Highlights •    Application of a novel multi-actor framework

•    Successful implementation of 9x FSLL

•    1000+ food-related indicator inventory

•    Application of numerous tools

•    Demonstrated governance advancements

•    Novel knowledge exchange framework

•    Interactive Food System toolkit

## Declarations of interest

The views expressed in this article are solely those of the authors. Publication in Open Research Europe does not imply endorsement

## Declaration of Generative AI and AI-assisted technologies in the writing process

During the preparation of this work, the authors used OpenAI. (2023). ChatGPT (August 3 Version) [Large language model]. https://chat.openai.com, in order to correct sentence structure and grammatical errors. After using this tool, the authors reviewed and edited proposed changes and take full responsibility for the content of the publication.

## Introduction

### Food System transformation

Food Systems encompass interconnected socio-economic factors including the production, processing, distribution, preparation, consumption, and management of food, as well as the interactions with the environment, people, resources, processes, infrastructure, organisations, and civil society at large ( HLPE, 2017). A transformation of European Food Systems considers each of these key aspects both individually and collectively enacting a shift to more sustainable practices ( Béné, 2020).

Despite significant progress in reducing global hunger, millions of people still suffer from food insecurity and malnutrition ( FAO *et al.,* 2020). And despite progress being made on reducing consumption of high-carbon foods, the associated impacts remain high ( EC DG AGRI, 2021; FAO, 2017; FAO, 2022). Moreover, Food Systems remain under stress in order to meet the demand of nutritious and affordable food of a global population ( FAO, 2022). Additional stresses are further exacerbated by current linear economic models, the Food System has a range of substantial environmental impacts, such as deforestation, greenhouse gas emissions, water pollution, and excessive use of natural resources ( IPBES, 2019). Industrial agriculture practices make agriculture the leading cause of biodiversity loss and ecosystem degradation in Europe ( EEA, 2020; Foley *et al.,* 2005). Food Systems account for up to one-third of global greenhouse gas emissions ( Crippa *et al.,* 2021). There is a rise in diet-related diseases such as obesity, diabetes, and heart disease ( Willett *et al.,* 2019), and poor diet can be considered the leading cause of death globally ( GBD Diet Collaborators, 2019). Current Food Systems often perpetuate social and economic inequalities ( De Schutter, 2014), including a failure to account for large negative externalities ( Fan, 2021). Moreover, the hidden cost of food globally is 10 trillion purchasing power parity dollars according to the latest True-Cost Accounting estimate ( FAO, 2023).

The imperative for a Food System Transformation, as substantiated by Augustin *et al.* (2021) and Webb *et al.* (2020), is indisputable and encompasses objectives such as facilitating the consumption of sustainable meals ( Godfray *et al.,* 2018; Willett *et al.,* 2019) and upholding the fundamental right to food by ensuring safe, equitable access to adequate and nutritious food sources ( De Schutter, 2014; FAO, 2022). Furthermore, there is substantial potential to decrease GHG emissions in agriculture by shifting away from a predominantly livestock and animal feed-oriented system toward more plant-based food for human consumption ( Prag & Henriksen, 2020). A more Sustainable Food System would help mitigate environmental impacts, promote biodiversity, and address climate change while simultaneously improving resilience, adaptability, and food security ( IPBES, 2019). It would also address issues related to labour rights, fair trade, and gender equity, and support local Food Systems, enhancing social justice and equity ( De Schutter, 2014), as well as spanning cultural and health-related dimensions ( Moragues-Faus *et al.,* 2020). Such a societal-wide food system transformation would also need to address societal-wide challenges, including corporate influence over food-related policy decisions and institutional power imbalances ( IPES-Food, 2023), market, institutional, and policy failures perpetuating the instability of agrifood systems ( FAO, 2023), and a profit-driven incentive structure leading to runaway wealth inequality and carbon emissions inequality, whereby the super-rich 1% emit the equivalent of 66% of the global population ( Khalfan *et al.,* 2023).

To address these challenges, an effective transformation of the Food System will require a cross-sectoral and holistic approach ( Augustin *et al.,* 2021; FAO, 2018), achieving systemic intermediation that facilitates both horizontal and vertical interactions ( den Boer *et al.,* 2023). This encompasses sustainable agriculture practices, regional supply chains, reduced food waste, increased collaboration among stakeholders at local, regional, and global levels ( Ruben *et al.,* 2021), as well as system innovation integrating governance, social inclusion, product development, and processing ( Wascher *et al.,* 2015). Such a holistic transformation of the Food System will require degrees of decentralisationto redistribute value and knowledge ensuring the specific needs of local communities are met ( Moragues-Faus, 2020). City-region Food Systems offer a promising catalyst for change, demonstrating practical and scalable solutions that can reshape the Food System towards sustainability, resilience, and social equity ( Forster *et al.,* 2015).

The city-region Food Systems approach pioneered by the Milan Urban Food Policy Pact highlights the important role cities can play in transformation, including but not limited to facilitating collaboration across cities, developing urban food policies, reorienting school meal programmes, and promoting participatory actions ( Blay-Palmer *et al.,* 2018; Forster *et al.,* 2015). Cities are uniquely positioned to bring a place-based approach to food policy frameworks and foster local agency for change ( Moragues-Faus & Sonnino, 2018).

Furthermore, Living Labs (LLs) provide a good base for building multi-stakeholder initiatives and can be applied in many configurations, including city-regions, rural, and urban communities of practice. Living Labs form an integral part of future Food Systems through the promotion of research and innovation in food and nutrition security ( EC DG RTD, 2021), as well as offering a platform to increase citizen participation and implement policy change ( Brons *et al.,* 2022).

Living labs function as collaborative spaces where diverse stakeholders co-create and experiment with solutions to complex challenges ( Bouwma *et al.,* 2022). Food System Living Labs involve key actors, including farmers and local communities, who contribute to the development of context-specific solutions ( Yousefi & Ewert, 2023). By incorporating external mentors from academia, industry, and the local community, Living Labs foster knowledge exchange and skill development, driving systemic change ( Kok *et al.,* 2023). Utilizing schools as bases for these labs can also enhance student learning, empowering them to address real-world problems ( Chapagain & Mikkelsen, 2023).

Despite their potential, Living Labs face several challenges, including difficulties in measuring impact due to a lack of standardized evaluation frameworks and clear, quantifiable outcomes ( Erisman *et al.,* 2024; Yousefi & Ewert, 2023). Power dynamics within labs can also skew outcomes, as stakeholder relationships influence whose voices are prioritized ( Erisman *et al.,* 2024). Many labs are constrained by short-term projects, limiting their ability to generate long-term, systemic change ( Erisman *et al.,* 2024; Wascher *et al.,* 2023). Scaling successful practices to broader regions or contexts remains a significant challenge due to the complexity and uniqueness of each lab’s environment ( McPhee *et al.,* 2021).

To address these issues, further research is needed to understand how successful Living Lab models can be scaled and supported by policy frameworks ( Yousefi & Ewert, 2023). Encouraging interdisciplinary collaboration across fields such as social sciences, agronomy, and technology could lead to more comprehensive solutions. Additionally, extending project timelines and developing structured approaches to managing multi-actor collaboration would support sustained, systemic impacts ( Chapagain & Mikkelsen, 2023; Wascher *et al.,* 2023). Nevertheless, incorporating place-based Living Labs and their stakeholders with overarching high-level directives offers a framework to facilitate systemic intermediation leading to Food Systems change.

### Objectives

The objectives of this paper are: 1) to describe how multi-actor networks utilising Living Labs can drive Sustainable Food System transformation in Europe; 2) to document the nine Food System Living Labs as use cases; 3) to summarise best practices and common challenges of interdisciplinary collaborations; and 4) to reflect on and highlight how this approach can add value to more Sustainable Food Systems.

### Implementing the FoodSHIFT 2030 Project

**Figure 1.**
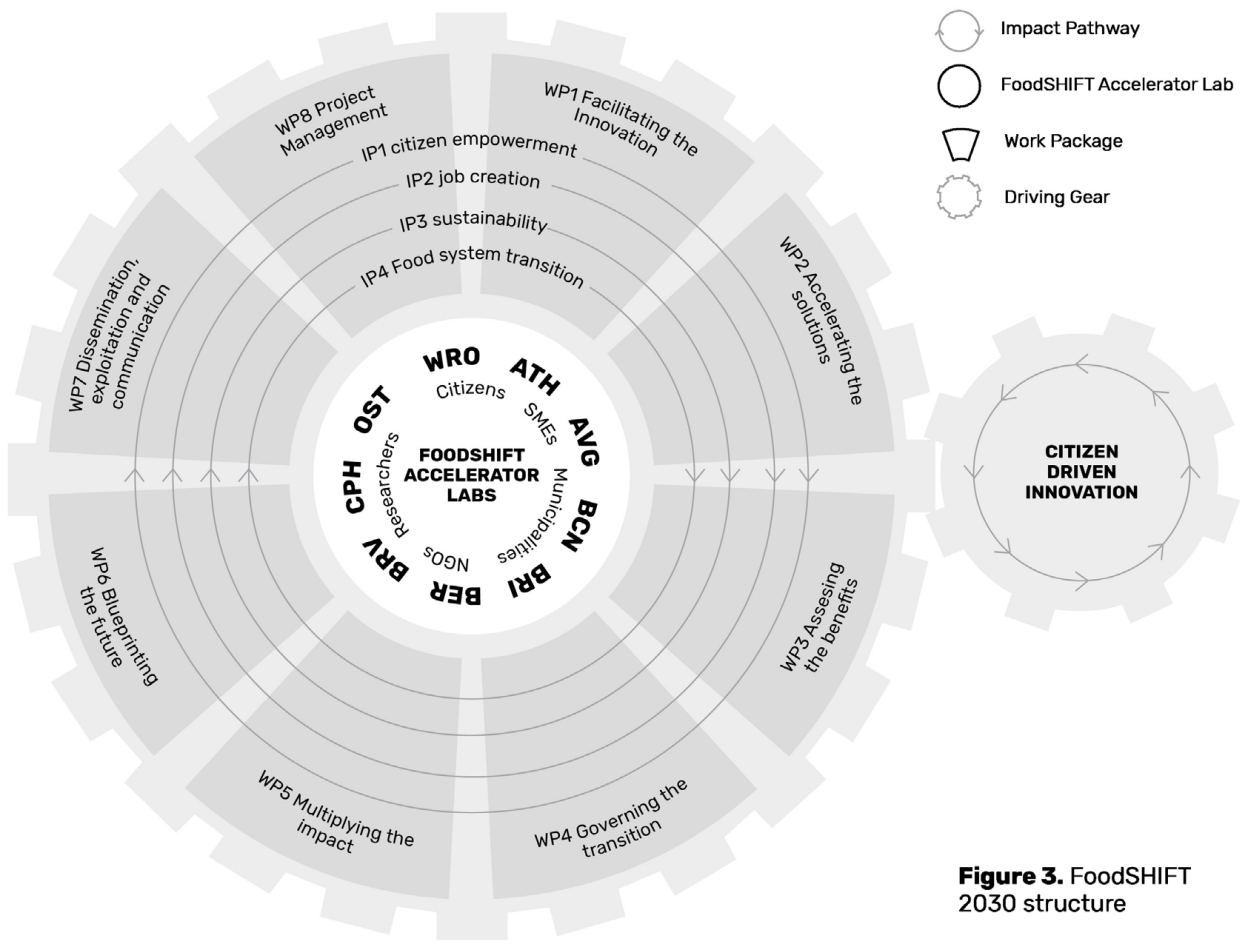
Visualising the FoodSHIFT Approach and how the Work Packages (WPs), Impact Pathways (IPs) and the nine FSLLs as FoodSHIFT Accelerator Living Labs interact with one and other (ATH = Athens GR; AVG = Avignon FR; BCN = Barcelona, ES; BRI = Bari, IT; BER = Berlin, DE; BRV = Brasov, RO; CPH = Copenhagen, DK; OST = Ostend, BE; WRO = Wroclaw, PO), © FoodSHIFT, 2019.

The FoodSHIFT Approach is a novel method for putting social, technological, and governance innovation at the heart of the European Food System transformation, involving large cross-disciplinary multi-actor networks. The FoodSHIFT Approach is outlined in Figure 1 and can be summarised into three main components: i) work packages with diverse expertise that combine the overarching goals of the EU Commission’s Green Deal, Farm to Fork Strategy, and FOOD 2030 pathways ( EC, 2019; EC DG SANTE, 2020; EC DG RTD, 2020); ii) FoodSHIFT Accelerator Labs, or Food System Living Labs (FSLLs), operating at the grassroots level; and iii) Impact Pathways that operate tangentially across all work packages and Living Labs, ensuring that key strategic goals remain in focus during every action.

The FSLLs utilise clear roles and divisions of labour, fostering the structure to adequately manage the multi-actor FSLL. The FSLL management core group usually included a Lab Host representing local government, a Lab Leader usually an SME or NGO driving innovation, and a Lab Assistant, usually a research partner. The Lab Leader managed the FSLL budget and ensures participant motivation and progress, while the Lab Host provided the institutional setting and links to local governance. The Lab Assistant supportsed the innovation process and links to leading research. FSLLs independently defined their own focus and specific themes that align with Sustainable Development Goals (SDGs). The FSLL initiatives were operationalised through workshops and events, collectively working towards transformative change in the Food System ( Armstrong *et al.,* 2020). The FoodSHIFT FSLLs also engaged a broader steering group composed of key local stakeholders representing various sectors, including the private sector, public sector, voluntary sector, and academia. Similar leadership structures, such as the UK’s Sustainable Food Places, have delivered knowledge and agency to diverse food actors and empowered change through trans-local initiatives ( Moragues-Faus & Sonnino, 2018).

The nine FoodSHIFT FSLLs developed their own goals from the bottom up based on their expertise and their Sustainable Food System innovation aspirations. The Open School Lab in Athens reconnects young people with the land, promotes healthy eating and plant-based foods, and finds solutions for using leftovers in school canteens. The Regional Lunch for All in Avignon uses public procurement to create a regional, sustainable, and healthy Food System by increasing local and organic food in school canteens and improving access to fresh food. The Food Tech 3.0 Lab in Barcelona develops open-source food technology for efficient urban food production and collaborates with FAB LAB initiatives. The FoodLifeCentre in Berlin establishes an urban food hub prototype, empowers local communities, and enhances accessibility to new infrastructures and knowledge. The Back to the Land Lab in Bari focuses on sustainable land use, short food supply chains, and youth employment through targeted plans. The Interactive Food Lab in Brasov integrates traditional and local producers, promotes local food innovation, and improves public policies and marketing. The Kitchen of Tomorrow Lab in Greater Copenhagen combines Food System innovations to provide sustainable and diversified local food, enhances connections between urban and rural areas, fosters the development of upcycled food, and implements climate-friendly food policies. The City Agro-Park in Ostend operationalises an agricultural park, strengthens the link between local producers and the catering sector, and supports education on local products and short food supply chains. The Accessible Food Gardens Lab in Wroclaw strengthens the local sustainable Food System through increased accessibility to food gardens, establishment of social gardens, engagement of young generations in urban farming, and creation of innovative community gardens for replication ( FoodSHIFT FSLLs, 2023).

Each FoodSHIFT FSLL together with support from specialised expertise from the work packages: (i) explored existing Food System innovations and develop tailored trajectories for further innovation, (ii) matured, combined, and up-scaled innovations while setting strategic targets and creating new business plans, (iii) assessed innovation ecosystems using co-developed indicators, evaluating their economic, environmental, and societal impacts, (iv) co-create governance strategies to support innovation and democratise Food System governance, and (v) multiplied its impact through a knowledge transfer ecosystem, enabling the exchange of knowledge and experiences between other initiatives and city regions worldwide ( Armstrong *et al.,* 2020).

Finally, four pathways to impact are: (a) The Citizen Empowerment Scheme which aimed to involve citizens in all aspects of the project and promote citizen-driven innovation and participation, (b) The Job Creation Platform sought to integrate job creation into the project's activities, including the development of implementation targets and business plans, (c) The Sustainability Scoring Scheme aimed to integrate relevant Sustainable Development Goals (SDGs) into the project's activities and assess the impacts of Food System innovations on economic, environmental, and societal sustainability, and (d) The Toolkit ( https://www.foodshifttoolkit.eu) provided comprehensive resources and information on Food System innovation, citizen empowerment, job creation, governance, and sustainability to guide stakeholders such as SMEs, NGOs, municipalities, and citizens ( Armstrong *et al.,* 2020).

## Food System Living Labs as use cases

Table 1– Table 9 outline specific use cases of nine front-runner FSLLs and their focus, achievements, core management group, local stakeholders and follower city-regions.

**Table 1.** The Open School Lab, Greater Athens, GR. *Schools as sites of food experience and Sustainable Food System transformation*.

***Leader:** Ellinogermaniki Agogi* ***Host**: Dimos Pallinis* ***Assistant**: DRAXIS* **7 Connected Local Innovators:** *Hellenic Society for the Protection of Nature,* *Eco-Schools, InCommOn, National Technical* *University Athens, SEVT, Organization Earth,* *Boroume and Future Intelligence* **5 Connected Follower FSLLs:** *Vrilissia (GR), Nea Smyrni(GR), Korydallos(GR),* *Halandri(GR), Agia Paraskevi (GR)*	**Key achievements include:** - An online community of teachers from around Europe on the SALL portal highlighted more than 60 educational projects following the Living Lab approach. - Two FoodSHIFT 2030 educational scenario contests and Teachers' Training Summer Schools were successfully hosted, promoting healthy eating and open innovation in education and health, following the Living Lab approach. - Open Schools Journal for Open Science showcased contributions from the Athens FSLL. - Development of food-related educational projects in schools. - EA school students engaged with the land through the school garden and visits to the Organization Earth urban garden, producers, etc. - EA became a partner of the European Partnership for a sustainable future of food systems, a long-lasting European endeavour, and the Urban Agenda for the EU Partnership on Food. - EA became a member of the Food Saving Alliance and has built a strong connection to the Ministry of Environment.

**Table 2.** Regional Lunch for All, Avignon, FR. *Public procurement as a driver for more regional, sustainable and healthy food*.

***Leader:** COMMUNE D'AVIGNON* ***Assistant:** INRAE* **10 Connected Local Innovators:** *Le CFA, Les Jardins de Solenes, de En* *Direct de nos Fermes, Supermarche* *Solidaire,* *Le Tipi, Le Tapy, Des Pieds et des Mains,* *Agrilocal, Local en Bocal, Les Alchimistes* **3 Connected Follower FSLLs:** *Albi (FR), Bergamo (IT), Bordeaux (FR)*	**Key achievements include:** - Implementation of a 6-month trial to compost the biowaste from the central kitchen, aiming to reduce food waste and explore sustainable waste management options. - Successful elimination of plastic usage, including the initiation of a partnership with an industrial washer in Avignon that facilitated the transition to sustainable containers. - Introduction of a biowaste collection and composting process for the central kitchen, contributing to waste reduction efforts. Ongoing efforts were made to find a suitable solution for the remaining 36 canteens. - Establishment of a partnership with the "Fair Supermarket" to define a donation process for leftover food, ensuring that excess food is redirected to those in need, reducing waste and supporting the community. - Serving as inspiration and a partner for more than a dozen other French cities to work on similar objectives, especially the elimination of plastic. - Raising awareness among key personnel to actively support the changes.

**Table 3.** Food Tech 3.0, Barcelona, ES. *A vision for food technology which places citizens at the center*.

***Leader:** Fab Lab Barcelona* **10 Connected Local Innovators:** *Look Ma’ No Hands, POWAR, ClosKa, Vilagreens, Ma!* *Condimentos vivos de Asia, Tectum Garden, NUMID, Domingo* *Club, Solo Aceite, Gaia Espirulina* **3 Connected Follower FSLLs:** *ARS longa. - Fab City grand Paris (FR), Open Dot. - Fab Lab Milan* *(IT), Fab City Hamburg (DE)*	**Key achievements include:** - Co-creation of a food technology vision with multi-actors of the local food ecosystem, using the Fab Lab approach to technology and social innovation. - Launched the Food Tech 3.0 acceleration program, supporting 10 citizen initiatives and creating the Food Tech 3.0 Gitbook. - Co-organized the Stakeholder Innovation Conference in Barcelona as part of its designation as the World Sustainable Food Capital for 2021. - Presented the FSLL Showcase de Barcelona, highlighting the progress of Food Tech 3.0 Acceleration Program initiatives.

**Table 4.** Back to Land, Bari, IT. *Sustainable land use and food chain strategies for young entrepreneurs building on social innovation in food systems*.

***Leader:** CIHEAM Bari* ***Host:** Città Metropolitana di Bari* ***Assistant:** Università degli Studi di Milano* **10 Connected Local Innovators:** *Synapsis, Cooperativa Sociale Siloe, Social Lab, Bio-* *Distretto delle Lame, Tracceverdi, Rete Buono e Bio,* *Semi di Vita, Avanzi Popolo, Artemisia, Orto Domingo* **3 Connected Follower FSLLs:** *Tirana (AlI), Taranto (IT), Tricase (IT)*	**Key achievements include:** - Co-created the Food Manifesto for the Metropolitan City of Bari, guiding its Food System transformation based on 9 core principles. - The Manifesto served as the primary reference for the city's food policy action plan. The latter was approved on September 28, 2023, for inclusion in the Metropolitan City's Strategic Plan, reaching 41 municipalities in the region. - The FSLL project supported innovators and initiatives via CIBA2030.it, fostering visibility and networking. - The Taranto Fellow city-region developed an urban food policy strategic plan based on the Bari Food Manifesto. - CIHEAM Bari is scaling the FSLL approach in the Mediterranean to support southern Mediterranean cities.

**Table 5.** FoodLifeCentre, Berlin, DE. *An innovation hub for sustainable regional food supply based on a decentralisation concept for food distribution and education*.

***Leader:** Baumhaus* ***Host:** Ernahrungsrat Berlin E.V.* ***Assistant:** ZALF* ***Assistant:*** *agrathaer GmbH* **10 Connected Local Innovators:** *Restlos Glücklich, PlantAge, Querfeld,* *Gemüse Syndikat, Biokräuterei* *Oberhavel, Supercoop Berlin,Wo* *kommt dein Essen her?, Food Hub* *(LMP) Network* **3 Connected Follower FSLLs:** *Lübeck (DE), Bochum (DE), Zürich* *(CH), Freiburg (DE)*	**Key achievements include:** - Baumhaus established the Urban Food Hub Prototype and expanded it to different districts. - BFPC provided political support for civic-driven actions, while ZALF conducted participatory research for impact assessment. - Ongoing engagement included meetings, workshops, and support for LMP initiatives such as aiding a farm with digital tools. - LMP Activations, "LMP Aktionswochen 2021" and "LMP Mitmachaktionen 2022," featured public events, volunteer sessions, and over 160 events across Berlin districts. - The movie "Seeds of Change" gained recognition from the Federal Agency for the Protection of Minors in the Media. - Engaged with the German Parliament, regional Food System workshops, and district-level food policy strategy development. - The food hubs were included in the Berlin municipal coalition agreement. - Received major additional funding for the food hub network, allowing it to scale from three initiatives in 2020 to 27 in 2023, and still growing. - Building trust and networks among Berlin Food System actors catalysed the ecosystem.

**Table 6.** Interactive Food Lab, Brasov, RO. *Integrating traditional and local producers into an innovative and ambitious regional Food System*.

***Leader:** Highclere Consulting* ***Host:** Metropolitan Agency for* *Sustanable Development Brasov* ***Assistant:** Iceberg* **8 Connected Local Innovators:** *SolBun Cooperative, Local Action Groups* *brought together in a Living Lab, Small-* *local producers, Chef Daniela Graură* *on Sustainable Gastronomy Guide,* *Chef Mădălina Roman on reducing* *food waste, Local Gastronomic Points,* *Greening the Local Public Procurement,* **3 Connected Follower FSLLs:** *Alba Iulia City Hall (RO), Sibiu County* *Council (RO), University of Agricultural* *Sciences and Veterinary of Cluj-Napoca* *(RO)*	**Key achievements include:** - Engaged with local stakeholders, including the Breasla Cârciumarilor Association, which promoted a local dessert and developed a direct relationship with local food suppliers. The SolBun cooperative sold local products in the Star Market and organized tasting sessions to strengthen relations with consumers. - Collaborated with a local expert to analyze public procurement legislation regarding the ‘Bread and Milk in schools’ program and sent recommendations for improving the program to the Ministry of Agriculture and Rural Development. - Began developing a strategy to document the Food System in Brasov and created a structure for the sustainable gastronomy guide. - Raised awareness among public authorities about the importance of a SFS. - Promoted short-supply chains and coordinated collaborations between the SolBun cooperative and local gastronomic points and restaurants to organize events promoting local products and reducing food waste. - Integrated the food aspect into the strategies of Local Action Groups and applied for training sessions to professionalize small producers in Brasov County. - Developed a construction plan and began implementing an educational urban garden project.

**Table 7.** Kitchen of Tomorrow, Greater Copenhagen, DK. *Public procurement and professional kitchens for a sustainable regional Food System, including rural and coastal areas*.

**Leader:** *Changing Food* **Assistant:** * Circular Food Technology* **Assistant:** University of Copenhagen **9 Connected Local Innovators:** *Bowline, Changing Food,* *Madfællesskabet, Coop Crowdfunding,* *CPH Foodspace,* *Grønt Marked, Madland, Pantrii* *(Rahandel), Dagens Farm, Foodprint* *Nordic, Agrain* **3 Connected Follower FSLLs:** *Basel (CH), Kaunas (LI), Plymouth-Devon* *(UK)*	**Key achievements include:** - Citizen engagement via the ‘Choose Your Own Adventure Canteen Campaign' implemented at the University of Copenhagen. - Participated in the Grasp festival, collaborating with Bowline, Farmers, Copenhagen Business School, and restaurants to promote dialogue and connections in the Food System. - Joined the kick-off event of "Our Plant-based Future" at the Green Solutions Center (GSC), supporting cross-disciplinary student projects for a transformation to a plant-based Food System. - Engaged in events focused on social factors and barriers for climate-friendly food, particularly supporting plant-based food habits, including a KU and Concito event and the Parabere Forum. - Collaborated with Kitchen Collective, participating in various events and initiatives together. - Developed and launched a tool to analyze procurement data on a range of indicators ( Schafer *et al.*, 2023b, in review). The tool has already been successfully used in a multitude of kitchens in Denmark and beyond.

**Table 8.** City Agro-Park, Ostend, BE. *Operationalisation of an agricultural park*.

**Leader:** *Stad Oostende* **Assistant:** *ILVO* **8 Connected Local Innovators:** *Agropark Gardens of Stene,* *Buitengoed, Sint Andreas* *Secondary School, Antenne* *and Food Savers, Duinhelm,* *Chef on the Move, Co-housing* *Boldersdorf, Ter Doorn,* *Tourisme Oostende, Cultuur* *Cafe* **5 Connected Follower FSLLs:** *Brugge (BE), Kortrijk, (BE), Eeklo* *(BE), Manger Demain (FR),* *Lisbon (PT)*	**Key achievements include:** - Signed the Milan Urban Food Policy Pact and the Flemish Green Deal Protein Shift. - Strengthened and linked community-supported agriculture (CSA) with the Agricultural Park, reaching 300 members, and further increased markets for local producers with a new farm shop and a bi-weekly farmer's market. - The Gardens of Stene conducted 20 outreach-education sessions on sustainable food production. - The central kitchen in Ostend achieved a 40% reduction in food waste, receiving the Foodwin Food Waste Award in 2022. - Twenty-three restaurants participated in the Veggie Challenge, promoting plant-based diets in 2022. - The Food Savers initiative gathered 25 tons of excess food monthly and provided food aid to 5% of the region's inhabitants, earning a nomination for the European Innovation Social Services Award. - The Food Savers initiative created 2.5 new jobs and offered 9 reintegrating learning places. - The Foodshift Food Lab developed 10 pop-up local recipes using excess food, conducted four Food Waste workshops, and hosted two food labs showcasing local products and entrepreneurs. - The food strategy was implemented, anchoring the topic of food in the city of Ostend long-term.

**Table 9.** Accessible Food Gardens, Wroclaw, PL. *Strengthening of innovation potential of local sustainable Food System*.

**Leader:** *Fundacja Ekorozwoju* **Host:** *Wroclaw Miasto* **Assistant:** *Uniwersytet Przyrodniczy We Wroclawiu* **10 Connected Local Innovators:** * Beekeeping Foundation, Foodsharing, Earth Market,* *Olbin Open Garden, TAJFUN, Healthy Seeds, The RAFT,* *Apple Reserve, From Hunger, Short Distance Bazaar,* *Food Think Tank* **4 Connected Follower FSLLs:** *Krzyżowa (PL), Manchester (UK), Trento (IT), Warsaw (PL),*	**Key achievements include:** - The city signed the Milan Urban Food Policy Pact, showcasing its commitment to sustainable food policies. - Twenty-five out of the initially planned four vegetable gardens were created at schools and kindergartens, promoting education and access to fresh produce. - WroCHEF, a culinary competition for schoolchildren, was organized in collaboration with the Wrocław Council for Gastronomy and restaurateurs to promote plant-based dishes. - Workshops were conducted at various levels of involvement to raise awareness and educate the community. - Collaboration with schools and community groups led to the sharing of ideas and the establishment of community gardens through diploma thesis projects and other funding opportunities. - Established a community-supported agriculture (CSA) with 100 members from the university.

## Project outcomes

The FoodSHIFT Approach has intensified interactions among all actors in the food chain, connecting over 150 multi-disciplinary organizations across 40 city-regions in 18 countries. Additionally, it has demonstrated the empowerment of local communities by combining high-level policies with grassroots change makers via the FSLLs. The multi-actor collaboration has fostered long-term positive economic, social, and environmental links between urban, peri-urban, and rural areas, built on the strong foundations of the FSLLs that continue to expand and engage Food System actors in local and European arenas.

### Establishing Food System Living Labs

The establishment of the nine front-runner Food System Living Labs (FSLLs) encompassed the development of guidelines and organizational structures to define roles and establish relationships within the project structure. The full guidelines are made available as a project deliverable ( Prove *et al.,* 2020). All lab members involved received a series of training workshops on open innovation and participatory learning and action, which equipped FSLLs with tools and approaches for stakeholder mapping, priority setting, and group facilitation techniques ( Brouwer & Hessels, 2019). Each FSLL directly engaged with a maximum of 10 local Food System innovators and employed a methodology to gather qualitative and quantitative information using two tools: MICRA and an online Lime Survey. Through participatory workshops and discussions, each FSLL established common priorities to enhance stakeholder engagement, stimulate ownership, and promote collaborative efforts. Additionally, each FSLL co-developed a novel approach, the Tailor-Made Trajectories (TMTs), to align objectives with innovation focuses and lab impacts, ensuring alignment of works throughout the project.

### Food innovation ecosystem

Building directly on the established FSLL base, preliminary food innovator information, and tailor-made trajectories, each FSLL then activated its regional Food System innovation ecosystem. In direct collaboration with their local innovators, each FSLL determined a baseline innovation readiness level (IRL) for each innovator to be monitored throughout the project. An Innovation Report and Food Ecosystem Roadmap were created to present innovators according to a mixture of metrics such as maturing, combining, and upscaling. Reaching similar conclusions as other Living Lab-focused studies ( Kok *et al.,* 2023), it was clear that conventional output-oriented evaluation methods alone were insufficient to capture the socio-cultural changes. As such, novel methodologies were co-created with FSLLs to measure and record Food System innovation on a city-region network level by utilizing:

a) The Helicopter view – capturing acceleration activities and outcomes on a systemic level in the city-region, including IRL progress of the 10 innovators.
b) The Deep Dive – capturing best examples of acceleration at the innovator level (case-study format).
c) Checkpoints – monitoring FSLL innovation progress on a six-week basis and producing a steady stream of status reports monitoring the IRL increases of innovators as well as good practices according to the innovation focus. The checkpoints also enabled the identification of barriers for Food System transformation, as well as identifying opportunities for cross-pollination between FSLLs.
d) Innovation Readiness Levels – development of a broader set of Innovation Readiness Levels as an alternative to Technological and Societal Readiness Levels. The IRLs describe the level of maturity of innovation in a broader sense, ensuring that innovations that do not fall into the traditional definitions of technological and societal innovations can also be captured for monitoring purposes ( Wascher *et al.,* 2022).
e) Additional support included three targeted business plan development workshops conducted with innovators across Europe. Finally, public-facing Summary Factsheets were created, detailing lessons learned and main acceleration outcomes across the FSLLs.

Factsheets depicting some of the key findings are made available as a project deliverable ( Eyre *et al.,* 2022).

### Assessing the Food System

Alongside the activation of local food innovation ecosystems, targeted efforts were made to quantitatively and qualitatively assess the benefits of diverse Food System innovators and the innovation ecosystem as a whole. A unique template for integrated, standardized, comparable data collection and assessment was created to support data collection based on the FAIR (findable, accessible, interoperable, and reusable) principles. Next, an extensive literature review of existing indicators and frameworks ( Aslanoglu *et al.,* 2023, in review) was performed with emphasis on gauging Food Systems across a large range of metrics including social, environmental, economic dimensions, as well as principles of Food and Nutrition Security and Food Sovereignty. This resulted in a meticulously sorted and pre-screened inventory of over 1000 Food System-related indicators for FSLLs to select from according to their unique innovation focus. The full indicator set is made available as a project deliverable ( Anthouli *et al.,* 2022). Furthermore, the current state of the Food Systems within each FSLL was evaluated using various approaches such as the Metropolitan Foodscape Planner (MFP) ( Arciniegas *et al.,* 2022), City-Region Foodshed Assessment (CRFA), and Metropolitan Foodshed and Self-sufficiency Scenario (MFSS) model ( Sylla *et al.,* 2022). Additionally, spatial models and scenarios were created to visualize the FSLL innovation ecosystem network and identifying key areas for development. All inputs were consolidated into an overarching assessment of the synergies and trade-offs based on agro-ecological principles ( Vicente-Vicente *et al.,* 2023).

### Advancing food governance

To advance Food System governance across various levels of society, specific steps were taken within the FoodSHIFT project. Recognizing the diversity within Living Labs, a new best practice framework for citizen-driven Food System governance was developed. This framework is made available as project deliverable ( Walthall *et al.,* 2022) and focused on four core dimensions:

a) Food as a Common Good: Emphasizing the importance of food as a shared resource that should be accessible to all members of society.
b) Inclusion of a Diverse Group of People: Ensuring that diverse voices and perspectives are represented in decision-making processes related to Food Systems.
c) Collective Actions and Advocacy: Encouraging collaborative efforts and advocacy initiatives aimed at addressing Food System challenges and promoting positive change.
d) Considering a Diversity of Knowledge: Acknowledging the value of various forms of knowledge, including traditional and local knowledge, scientific expertise, and experiential knowledge from community members.

This framework served as the basis for the development of unique food strategies in four FSLLs: Avignon, Bari, Brasov, and Berlin. Each of these strategies focused on different thematic areas, such as improving metropolitan procurement and catering systems, enabling the local Food System environment through governance innovation, strengthening connections between regional food economy, culture, and tourism, and improving regional food production.

Moreover, over 30 follower FSLLs were empowered to develop their own food strategies using newly developed Food System governance advisory factsheets. These factsheets provided guidance on key topics such as Food System governance, setting up a food strategy, and building up a Food Policy Council. Finally, the impacts and learnings from these efforts were accumulated and disseminated through workshops, conferences, and targeted policy briefs. This dissemination aimed to facilitate institutional learning and drive high-level Food System governance transformations at local, regional, and EU levels.

### Targeted knowledge exchange

All key learnings, tools, practices, factsheets, and findings were expertly exchanged throughout various networks and communities within the FoodSHIFT project. It began with the establishment of a dedicated framework facilitating peer-to-peer knowledge transfer among all nine front-runner FSLLs. This peer-to-peer exchange continued, supported by a Health Check survey aimed at better understanding the heterogeneous relationships between stakeholders, as well as their needs and potentials.

Expanding knowledge exchange further was the introduction of 33 follower FSLLs, each undergoing a unique application and screening process to join the internal network of FSLLs and work towards establishing their own FSLL. Continuous facilitation of peer-to-peer exchanges was carried out through various actions and initiatives, including numerous targeted webinars utilizing online brainstorming tools, individual interviews and recordings, and an online excel-based calendar to better forecast and provide equal opportunity within exchanges. Additionally, facilitation and encouragement of in-person networking events supplemented these efforts.

To guide, monitor, and maintain efficient and effective exchanges, novel Knowledge Brokerage Fact sheets were developed and made available as a project deliverable ( Beblek & Frühbeißer, 2022). Finally, targeted knowledge exchange was pursued beyond the immediate network with the intention of long-lasting impacts. This included a series of ten uniquely themed public-facing webinars featuring guest experts from around the world, four dedicated public workshops sharing food system learnings, and continuous connection offered by the public FoodSHIFT+ email exchange forum.

### Impacts pathways

Impact Pathways on fundamental objectives operated tangentially across all work packages and FSLLs to ensure core principles were considered in every action. These were the four Impact Pathways: Citizen Empowerment, Job Creation, Sustainability Scoring System, and the FoodSHIFT toolkit.

A Citizen Empowerment Scheme, made available as a project deliverable ( Crivits & Mingolla, 2022), was developed to empower citizens, particularly marginalized or vulnerable groups, to make informed decisions on sustainable food and increase participation in their regional Food System. Associated initiatives included collaboration with Citizen Lab, and the 'choose your own adventure canteen campaign' facilitated by Copenhagen FSLL ( Schafer *et al.,* 2023a, in review).

A blueprint for creating a decentralized job platform was developed based on practice-based applications is available as a project deliverable ( Corsi & Ruggeri, 2022), The blueprint included a functional front-end development prototype as well as a key focus on searchable sustainable food jobs, tailored and localized opportunities, efficient matching, community involvement, and regional adaptability.

The Sustainability Scoring System is a functional prototype designed to score heterogeneous Food System innovators against a set of sustainability principles derived from the FoodSHIFT indicator inventory. The SSS included recommendations on setting up a scoring committee, as well as guidelines for scorers and is available as a project deliverable ( Anthouli & Valta, 2022)

Finally, the FoodSHIFT Toolkit is the central hub where all outputs are indexed, serving as a legacy item for the sustainable food community. The toolkit is a web-based application containing a searchable collection of tools and resources designed to aid Food System stakeholders, including citizens, city representatives, innovators, and NGOs, to take action in their own city region. The development of a toolkit offers user-oriented support for stakeholders to search and sort key outputs to better understand, stimulate, and measure the transformation in their local Food Systems, including a validated framework for organizing Living Labs, several innovation assessment methodologies at the city region level, a directory of local food innovation cases, and real-life recounts from the Living Labs in the form of city-stories.

### Communications

A dedicated communications team developed a dissemination, exploitation, and communication plan, which included improvements to workflows and the creation of a value manifesto and templates. Developed brand assets, logos, and illustrations. Enhanced communications utilizing a content creation workflow, content calendar, value manifesto, and drag-and-drop templates. External communication included 22 webinars, 1 stakeholder conference, 1 policy conference, 4 press releases, 4 Instagram reels, 5 interview videos, 41 videos on the FoodSHIFT YouTube channel, and numerous tweets. Managed the project website and social media accounts, including new content, such as blog posts, embedded videos, deliverables, and scientific publications. Increased outreach and engagement with an audience of 58,849 at online/offline events, 122 events hosted/participated, 56,500 website views, 1,900 newsletter subscriptions, and 23 published newsletters.

### Management

A diverse management team oversaw the entire project, which included a coordinator, Innovation Manager, two Project Managers, EU Commission Liaison Officer, Financial Officer, Data, and Data Manager. This team maintained a proactive management style implementing many tools, procedures, and guides including facilitated executive board meetings and common decisions, managed online workspace planned common events, created an Interactive Project Gantt Chart, created a sortable contact list, and developed decision diagrams for internal navigations of Ethics and Data as well as useful tools being adopted by other projects such as continuous rolling agendas.

## Discussion

### Overarching objectives

The FoodSHIFT 2030 project has undoubtedly achieved significant success in meeting its stated objectives and impacts. Evaluation by European Commission Project Officers, impartial reviewers, and external innovation experts has consistently yielded positive remarks, highlighting the project's effectiveness and innovative approach. The FoodSHIFT Approach, in particular, has garnered praise for its groundbreaking nature and has been recognized as a good practice within various EU communities ( INTERREG, 2022).

Moreover, the FoodSHIFT Approach has effectively addressed the priorities outlined in the Food 2030 agenda, focusing on sustainable and healthy diets, environmentally Sustainable Food Systems, circular and resource-efficient practices, and fostering innovation through community empowerment. Similarly, it has aligned well with the objectives of the European Green Deal and the Farm to Fork strategy, promoting sustainable food production, consumption, and reducing food waste, among other key priorities.

FoodSHIFT2030 has substantially contributed to the transformation of Food Systems, particularly through the legacy of some of its most effective tools. The citizen engagement initiative, which effectively transforms citizens into proactive agents of change, magnifies the impact of single and local policies in wider contexts. The job platform promotes a collaborative alliance between employees and companies, all aimed towards the shared goal of transforming Food Systems. The Sustainability Scoring System provides a mechanism for quantitatively assessing the contributions of citizens, corporations, and public entities, steering them towards a collectively sustainable future. Complementing these is the toolkit, a web-based application comprising a comprehensive repository of tools and resources tailored to assist in transforming Food Systems. The toolkit democratizes access to a wealth of information, rendering it available to an extensive array of stakeholders including citizens, city officials, innovators, and NGOs. Additionally, the dissemination of knowledge through and between FALs and FELs initiates a cascading effect, anticipated to exponentially grow in the foreseeable future. This dynamic contributes to the enduring legacy of the FoodSHIFT2030 project, which is strategically positioned to foster sustained transformation within the food sector over an extended period.

FoodSHIFT members led the publication of a joint policy brief by five projects under the title of the EU FOOD 2030 Project Family that serves as a base for the following reflections and recommendations section. The five projects represented in the brief were FoodSHIFT 2030, FOODE, Food Trails, Cities 2030 and Fusilli. The brief outlines experience-based recommendations in order to achieve long-term support for Food System Living Labs towards Food System transformation ( Wascher *et al.*, 2023). More specifically, the brief draws attention to the importance for long-term institutional commitment to Living Labs as they offer an essential interface of cities/regions and the wider countryside. The brief also calls for paradigm shift away from linear value systems to a more circular, holistic approach to better capture true cost of food and ensure a fair transformation. The policy brief emphasizes the need for a timely and pro-active solution to secure co-founding for the local facilitators to ensure there is a post-project continuation of works from the many successful Food System Living Labs across Europe,

It is important to recognize that achieving cross-sector European Food System transformation requires a broader and long-term approach beyond individual projects. While FoodSHIFT 2030 has made significant strides, broader systemic change necessitates collaboration across sectors and policy instruments. Critical reflections are essential to identify areas for improvement and prospects for leveraging the principles and experiences of FoodSHIFT towards a more comprehensive Food System transformation. 

Furthermore, the FoodSHIFT findings would benefit from a gap analysis that highlights areas that were either insufficiently detailed or excluded as beyond the project's scope. Most notably, the impact that transforming Europe's Food Systems would have on various parts of the global supply chain. Given the interconnected nature of global food supply chains, transformative actions in Europe would be best accompanied by equally transformative, well planned interventions conducted simultaneously across the entire food supply chain. This approach would maximize the benefits of sustainable Food Systems while minimizing potential negative effects, especially for producers in low-income, high-export countries.

In summary, while FoodSHIFT 2030 has demonstrated success in its objectives and impacts, it’s true legacy lies in how the approach and experiences can be used to inform broader efforts and avoid some of the difficulties encountered while implementing FoodSHIFT.

### Experienced Based Reflections and key areas for improvements

In developing a robust approach to the Food System, a multi-actor strategy is imperative, given the diversity of actors involved, each with unique needs and operational methods. To ensure effectiveness, establishing well-defined work plans and delegating responsibilities are crucial. Emphasis should be placed on collaboratively identifying and prioritizing common reward streams, fostering a shared vision that enables the exploitation of mutually beneficial targets. Furthermore, bridging the gap between food programs and citizen engagement initiatives is essential, necessitating a culture that views citizens as active change-agents. Efforts should intensify to persuade local public agencies to seamlessly integrate citizen engagement into their operations, fostering a more inclusive and participatory approach.

Supporting innovators within the Food System presents its own challenges, particularly when targeting individual experts who are often highly advanced in their fields. To overcome this, activities should transcend individual needs, providing more accessible and universally beneficial support. A project's flexibility to engage or disengage innovators over time aligns with the dynamic nature of innovation ecosystems, crucial for staying attuned to evolving innovations and addressing changing needs within the ecosystem.

Enhancing the effectiveness of a Food System transformation requires fostering integration among various stakeholders by identifying and aligning their mutual interests, encompassing both companies and workers. Going beyond the decentralized FoodSHIFT job platform, efforts should be directed towards creating specific pathways for upskilling and retraining existing labour force to match a job opportunities within a Sustainable Food System. Combining new employment pathways with decentralised job marketplaces that efficiently match workers with new roles that best suit their existing skills and competencies. Such an arrangement would facilitate the active involvement and empowerment of the labour force in achieving overarching business-policy objectives.

Crafting decision support for Food Systems demands a comprehensive approach to cater to diverse target groups, including citizens, innovators, and governance stakeholders. Moreover, any decision support tool is reliant upon data quality and comparability. Monitoring Food System innovation requires a lean and dynamic methodology, especially given the susceptibility of Food Systems to external forces and rapid changes. The adaptability and feasibility of the monitoring approach are paramount, ensuring it remains responsive to external influences while capturing key information. Anticipating various methods, and combinations of methods, to assess innovation levels and progress becomes pivotal. Much more foresight and investigation is needed to create effective monitoring metrics that best capture existing Societal, Technological and Innovation Readiness Levels alongside a governance readiness level or similar to capture the political-will behind an innovation. Combining these overarching metrics with adaptable quantitative and qualitative methods will provide an improved holistic perspective on the maturity and potential impact of innovations within the Food System.

### Reflections on the operationalisation of Food System Living Labs

Navigating the complex landscape of a multinational initiative focused on Food Systems transformation has unveiled several critical reflections pertinent to the FSLLs acting as catalysts for bottom-up change. A prominent challenge lies in overcoming language barriers and requires dedicated attention and resources to ensure engagement of local authorities from diverse countries. Another pivotal aspect to address is the delicate balance between providing targeted support for a plurality of needs which is inherently highly resource intensive and requires significant relations management to ensure all actions are relevant and beneficially for the intended audience. Achieving a common understanding of terminology, especially project-specific language, emerges as a priority. Establishing shared definitions for these terms is essential for fostering coherence and facilitating effective cross-border collaboration.

To enhance the initiative's impact, there is a call to directly include more mayors and elected officials into the works, allowing them to witness and comprehend the innovative strategies being implemented. Moreover, recognizing empowering the pivotal role of FSLL Leaders and the importance of co-funding options to ensure they can continue to be the center point for continuous progress on all works. Having FSLLs central to roundtable discussions enabling the presentation of their perspectives and city-specific challenges, while project stakeholders listen and strategize on how to provide support, can lead to the extraction of valuable knowledge and identification of commonalities across diverse contexts. Acknowledging the heterogeneous characteristics of the FSLLs and the varying contexts they operate within is crucial. Navigating solely via FSLL leaders poses potential bottlenecks to progress, and where possible leaders should broker direct connection between prominent experts and city-region stakeholders. Maintaining community momentum and relevance is challenging when political will is subject to change, which can result in periods of lower engagement. To counter this, ongoing initiatives should prioritize community outreach to remain responsive to evolving needs.

### Reflections on management, internal communications and EU projects

The third reflection section relates to management, project structure and EU funding. There is a need for increased financial flexibility in funding payments to better cater to the liquidity requirements of small local operations. Board Restructuring: Recognition of the ineffectiveness of a large management board (20+) prompts a board restructure to a more compact and strengthened decision-making authority as well as a simple mechanism for joint approvals for key works. Projects that deploy FSLL methods and focus on bottom-up approach should be certain to align deliverables directly with FSLLs' existing aims or offer universal benefits. Emphasis needs to be placed on strong coordination and leadership throughout large, complex projects to maintain focus on overarching goals. Recognising challenges of internal communication within a sizable team (160+) and recommendations include limiting emails, enhancing workflow synchronization, and proposing a 'relations manager/team' for central communication. A need to address the Stop-Start Nature of EU projects leading to suggestions for additional funds toward the end to counter the lengthy conceptualization period (12–24 months) for project partners. EU Calls with a developmental / technical focus are advised to allow extra subcontracting funds for key technical experts or to coordinate and limit the number of projects developing their own, often competing technical solutions. Finally, striking the balance between empowerment and quality outputs is emphasized in participatory methods, ensuring ample time and resources for scientific rigor while linking back to proven concrete principles

## Conclusion

The FoodSHIFT 2030 project ambitiously deployed a multi-actor collaboration aimed to support the transformation of the European Food System towards a low-carbon, circular future by place-based Living Labs harnessing and empowering citizens to-develop sustainable food solutions for the future. The FoodSHIFT Approach offers a functional framework utilising Living Labs as place-based community driven spaces to harness citizen empowerment and facilitate transdisciplinary workflows toward more Sustainable Food System. More research into key findings, particularly in assessing the long-term potential impact of citizen driven FSLLs, improved employment pathways to enable labour forces to transform with the Food System and the simultaneous transformative action to be deployed not just in Europe, but across global supply chains to deliver a more Sustainable Food System for more people. Nevertheless, the knowledge and experience gained in the FoodSHIFT approach is required reading for any future EU initiatives implementing Living Labs working towards a more Sustainable Food System.

## Data Availability

No data are associated with this article
