# Supplementary material for: Transforming European Food Systems with multi-actor networks and Living Labs through the FoodSHIFT Approach
Source: Open Res Eur. 2024 Oct 25;4:151. Originally published 2024 Jul 18. [Version 2] doi: 10.12688/openreseurope.17982.2 (PMC11535485; doi:10.12688/openreseurope.17982.2)
Supplement: Peer review history

# Reviewer report: Reviewer response for version 2

**DOI:** 10.21956/openreseurope.20289.r45503

**Published:** 2024-11-04

**Authors:** Tiffanie Stone

**Affiliations:** 1 Aarhus University, Aarhus, Denmark

Thank you for the thorough response. I am satisfied with the revisions. Congratulations and best regards,

 Tiffanie Stone

Where applicable, are recommendations and next steps explained clearly for others to follow? (Please consider whether others in the research community would be able to implement guidelines or recommendations and/or constructively engage in the debate)

Yes

Does the article adequately reference differing views and opinions?

Partly

Are all factual statements correct, and are statements and arguments made adequately supported by citations?

Yes

Is the rationale for the Open Letter provided in sufficient detail? (Please consider whether existing challenges in the field are outlined clearly and whether the purpose of the letter is explained)

Yes

Is the Open Letter written in accessible language? (Please consider whether all subject-specific terms, concepts and abbreviations are explained)

Partly

Reviewer Expertise:

I am a researcher in the Agroecology Department at Aarhus University. I study the social and environmental impacts of food systems at decision-making scales, food system transitions/transformations, FEWS nexus interactions, and landscape dynamics using mixed methods.

I confirm that I have read this submission and believe that I have an appropriate level of expertise to confirm that it is of an acceptable scientific standard.

# Reviewer report: Reviewer response for version 2

**DOI:** 10.21956/openreseurope.20289.r45504

**Published:** 2024-11-04

**Authors:** M.G van der Meij

**Affiliations:** 1 VU University, Amsterdam, The Netherlands

The manuscript has significantly improved, thank you for dealing with the peer comments so delicately.

Where applicable, are recommendations and next steps explained clearly for others to follow? (Please consider whether others in the research community would be able to implement guidelines or recommendations and/or constructively engage in the debate)

Yes

Does the article adequately reference differing views and opinions?

Partly

Are all factual statements correct, and are statements and arguments made adequately supported by citations?

Partly

Is the rationale for the Open Letter provided in sufficient detail? (Please consider whether existing challenges in the field are outlined clearly and whether the purpose of the letter is explained)

Partly

Is the Open Letter written in accessible language? (Please consider whether all subject-specific terms, concepts and abbreviations are explained)

Yes

Reviewer Expertise:

Transdisciplinary collaboration and co-creation; multi-stakeholder and citizen participation in system transformation

I confirm that I have read this submission and believe that I have an appropriate level of expertise to confirm that it is of an acceptable scientific standard.

# Reviewer report: Reviewer response for version 1

**DOI:** 10.21956/openreseurope.19436.r43842

**Published:** 2024-10-04

**Authors:** Tiffanie Stone

**Affiliations:** 1 Aarhus University, Aarhus, Denmark

This report provides a rich and interesting account of the FoodSHIFT 2030 project. However, some minor changes are needed to improve the conceptualization and the accessibility. 

**Does the article adequately reference differing views and opinions?**

 In the introduction, food system transformation is framed as mitigating environmental impacts and addressing social equity. My question is, would a food system transformation necessarily provide all of this? Maybe a holistic food system transformation or as you say later, an effective food system transformation would. I think the food system transformation frame is useful, but the introduction could more explicitly define and also reference differing views and opinions regarding food system transformation.

 Along similar lines, in figure 3, food system transition was the impact pathway when the frame in the introduction was related to transformation. Please clarify or modify the terminology in the figure to be consistent.

 There are likely many visions for a future food system, can you clarify this comment, who envisions this and are there any other relevant visions to include in this section: “The future food system is envisioned as decentralized, redistributing value and knowledge to ensure the specific needs of local communities are met ( Moragues-Faus, 2020).”

 Additional sources to support this claim would be helpful: “Living Labs form an integral part of future food systems through the promotion of research and innovation in food and nutrition security (EC DG RTD, 2021)”

**Is the Open Letter written in accessible language? (Please consider whether all subject-specific terms, concepts and abbreviations are explained)**

 In the abstract, both SME and NGO acronyms were used without description. In the case of NGOs, a wider group of actors may also be necessary for providing social innovation. MICRA was used in the results and INTERREG was used in the discussion without explanation.

 Figure 3: For acronyms within the FoodSHIFT Accelerator lab part of the figure. Please add a description to the caption.

 This is minor but please clarify the emission type: “and a profit-driven incentive structure leading to runaway wealth inequality and emissions inequality, whereby the super-rich 1% emit the equivalent of 66% of the global population ( Khalfan *et al.,* 2023).”

Where applicable, are recommendations and next steps explained clearly for others to follow? (Please consider whether others in the research community would be able to implement guidelines or recommendations and/or constructively engage in the debate)

Yes

Does the article adequately reference differing views and opinions?

Partly

Are all factual statements correct, and are statements and arguments made adequately supported by citations?

Yes

Is the rationale for the Open Letter provided in sufficient detail? (Please consider whether existing challenges in the field are outlined clearly and whether the purpose of the letter is explained)

Yes

Is the Open Letter written in accessible language? (Please consider whether all subject-specific terms, concepts and abbreviations are explained)

Partly

Reviewer Expertise:

I am a researcher in the Agroecology Department at Aarhus University. I study the social and environmental impacts of food systems at decision-making scales, food system transitions/transformations, FEWS nexus interactions, and landscape dynamics using mixed methods.

I confirm that I have read this submission and believe that I have an appropriate level of expertise to confirm that it is of an acceptable scientific standard, however I have significant reservations, as outlined above.

# Response

**Published:** 2024-10-14

**Authors:** Luke Schafer

**Affiliations:** Kobenhavns Universitet, Copenhagen, Capital Region of Denmark, Denmark

Dear Reviewer, We would like to sincerely thank you for your thoughtful and detailed feedback on our manuscript. Your insights and suggestions have been invaluable in helping us improve the clarity and quality of this manuscript. We have carefully considered all your comments and fully agree with the points raised. The manuscript has subsequently been revised to address each of them, copied below into corresponding sections with direct answers.   

Reviewer 2, Comment 1, Section - introduction: There are likely many visions for a future food system, can you clarify this comment, who envisions this and are there any other relevant visions to include in this section: “The future food system is envisioned as decentralized, redistributing value and knowledge to ensure the specific needs of local communities are met (Moragues-Faus, 2020).”

**Authors Response: **We agree that this statement should not stand alone as a singular vision but should find a more nuanced place within the broader context of systemic change. Therefore, we have rewritten it as follows: 'Such a holistic transformation of the food system will require degrees of decentralisation in redistributing value and knowledge to ensure the specific needs of local communities are met (Moragues-Faus, 2020).' Moreover, we agree and have removed the 'vision statemet' as it is subjective and baised based on the whose vision we are too look at. Instead, the manuscript still includes an elaborate list of components that could be part of a holistic, more sustainable food system.' Throughout the manuscript, we have carefully revised all instances of the phrase 'food system transformation' to ensure that it clearly describes the type of transformation being discussed, such as a shift toward more sustainable food systems. We have also removed the phrase in sections where specific actions were not mentioned."

Reviewer 2, Comment 2, Section - introduction: In the introduction, food system transformation is framed as mitigating environmental impacts and addressing social equity. My question is, would a food system transformation necessarily provide all of this? Maybe a holistic food system transformation or as you say later, an effective food system transformation would. I think the food system transformation frame is useful, but the introduction could more explicitly define and also reference differing views and opinions regarding food system transformation.

**Authors Response: **We agree that the framing of a transformation of a food system is important especially when considering the potential affects and societal-wide shifts that may arise from Food Systems change. And we completely agree that a food system transformation is exactly this systemic approach which is meant here to be synonymous with a holistic and effective transformation. the manuscript still includes an elaborate list of components that could be part of a holistic, more sustainable food system. Throughout the manuscript, any use of phrase 'food system transformation' has been carefully revised to ensure it is both described what kind of transformation, eg, moving toward more sustainable food systems, and has been removed in parts of the text not explicitly mention specific actions."

Reviewer 2, Comment 3, Section - introduction: Additional sources to support this claim would be helpful: “Living Labs form an integral part of future food systems through the promotion of research and innovation in food and nutrition security (EC DG RTD, 2021)”

**Authors Response: **We agree with both reviewers who highlighted the importance of this point, and we have heavily revised this section as Living Labs are a critical part of the manuscript. This includes adding four new academic references and rewriting two existing references to clarify their connections. These revisions are 3 full paragraphs now form the final part of the introduction.

Reviewer 2, Comment 4, Section - throughout: in figure 3, food system transition was the impact pathway when the frame in the introduction was related to transformation. Please clarify or modify the terminology in the figure to be consistent.

**Authors Response:** Agree and in general the project work recognises the need for a transformation and all uses of the word transition have been rechecked for appropriateness as well as either removing them, or, replacing with the word 'Transformation'.

Reviewer 2, Comment 5, Section - throughout: please clarify the emission type: “and a profit-driven incentive structure leading to runaway wealth inequality and emissions inequality, whereby the super-rich 1% emit the equivalent of 66% of the global population (Khalfan et al., 2023).”

**Authors Response: **Agree and have included carbon as the specific emissions mentioned here, and, have checked the manuscript for any other similar clarifications needed.

Reviewer 2, Comment 6, Section - throughout: In the abstract, both SME and NGO acronyms were used without description. In the case of NGOs, a wider group of actors may also be necessary for providing social innovation. MICRA was used in the results and INTERREG was used in the discussion without explanation. Figure 3: For acronyms within the FoodSHIFT Accelerator lab part of the figure. Please add a description to the caption.

**Authors Response: **Agree and have included at least the first inclusion of any abbreviation in full text throughout the manuscript

# Reviewer report: Reviewer response for version 1

**DOI:** 10.21956/openreseurope.19436.r42351

**Published:** 2024-09-04

**Authors:** M.G van der Meij

**Affiliations:** 1 VU University, Amsterdam, The Netherlands

The article outlines how the FoodSHIFT project has - according to the authors - successfully initiated / accelerated food system transformation through Living Labs in various cities throughout Europe. It summarises the approach that the project had adopted and what outcomes were achieved, and outlines what the adequate next steps should be to further accelerate food system transformation.

 As somebody who has worked in various EU projects - also on food system transformation - this manuscript is an interesting read and easy to follow. Considering the review criteria of Open Research Europe, I have a few (major) reservations, making me hesitant to consider the manuscript as it is now as suitable for indexing. I will proceed with my comments in the order of the review criteria 1. rationale of the manuscript, 2. different views, 3. citations/references.   

 When in comes to the rationale, it is not fully clear to me why this letter is structured as a regular scientific publication (intro, method, results, conclusion, discussion), while it is not a scientific research. This chosen structure makes it hard for a reader to understand what this article is about at first glance, and this chosen structure is also resulting in a manuscript that contains many redundancies (some things are being written twice or three times). Especially the method section - which describes the method of the project, not the method of scientific research into the project - is confusing in this regard. I would expect in a method section more explanation about how you came to the results that you present. The method/process of the project itself is more a contextual background thing, which can be added in the introduction about the project. To avoid redundancies, I would recommend restructuring the manuscript, and abandon the structure of a regular scientific manuscript.  

 In terms of different opinions, the manuscript merely presents FoodSHIFT as a 'super success' story. However, we all know that EU projects are very complex, very chaotic in the beginning, etc. so this multiple re-emphasis on how successful the project was is not something an EU project experienced reader may believe. In that sense, the authors could be more humble, and also share the struggles that they had, the insecurities they felt, the tensions between researchers and living lab practitioners (for example), etc., to accomplish the project results.

 Furthermore (in terms of different views), in the beginning of the manuscript there is quite a firm statement that there is a major role for the consumers to transform food systems by changing their diet. This comes across as little nuanced. It is known that consumers are subjected to the food system as it is now (capitalism driven), of which for example its massive advertisement (and low pricing strategy) for unhealthy food is highly influencing citizens. And, alternatively, citizens are not only consumers, they are citizens who can exercise different forms of power apart from consuming (think: producing, or activism, voting, etc.). I do see a note in the discussion (in 'actions and outputs toward food system transformation') on citizens being 'change agents'. I think it is wise to put this central in the introduction of the manuscript as well. Or be super self-critical on the project in case citizens were 'only' approached as consumers in the project. 

 Also, on page 4 the authors write about the need to decentralise the food system. Due to colonialism in the last centuries, many global food production and distribution networks were created, and when European cities decide to cut these up to and go local instead, major inequalities may arise. I would expect certain reflection on global inequalities that arise in the transition towards decentralised food systems (and how this was touched upon in the project). Maybe the authors did this in the discussion, but then I overlooked it. 

 In the manuscript, I miss certain references to other work at certain points. Foremost in the discussion section. But also in the introduction more references could be made to research into living labs. I do a few alternative suggestions that I know of below. 

 Last, with regard to the tables about the living labs in the manuscript, I would suggest putting the full names of the cities in there and not the abbreviations, or delete them in the tables and consider the caption as the best way to say which city/initiative each table describes (as you already do now). I understand that the project collaborators may have referred to one another in this way, but for outsiders ATH FSLL (for example) does not make much sense.  

 Good luck revising!

Where applicable, are recommendations and next steps explained clearly for others to follow? (Please consider whether others in the research community would be able to implement guidelines or recommendations and/or constructively engage in the debate)

Yes

Does the article adequately reference differing views and opinions?

Partly

Are all factual statements correct, and are statements and arguments made adequately supported by citations?

Partly

Is the rationale for the Open Letter provided in sufficient detail? (Please consider whether existing challenges in the field are outlined clearly and whether the purpose of the letter is explained)

Partly

Is the Open Letter written in accessible language? (Please consider whether all subject-specific terms, concepts and abbreviations are explained)

Yes

Reviewer Expertise:

Transdisciplinary collaboration and co-creation; multi-stakeholder and citizen participation in system transformation

I confirm that I have read this submission and believe that I have an appropriate level of expertise to confirm that it is of an acceptable scientific standard, however I have significant reservations, as outlined above.

## References

[1] : Labbing for sustainability transformations: Learning about challenges and strategies for impact. *GAIA - Ecological Perspectives for Science and Society* .2024;33(1) : 10.14512/gaia.33.S1.10 64-71 10.14512/gaia.33.S1.10

[2] : Exploring the practice of Labs for sustainable transformation: The challenge of ‘creating impact’. *Journal of Cleaner Production* .2023;388: 10.1016/j.jclepro.2023.135994 10.1016/j.jclepro.2023.135994

# Response

**Published:** 2024-10-14

**Authors:** Luke Schafer

**Affiliations:** Kobenhavns Universitet, Copenhagen, Capital Region of Denmark, Denmark

Dear Reviewer, We would like to sincerely thank you for your thoughtful and detailed feedback on our manuscript. Your insights and suggestions have been invaluable in helping us improve the clarity and quality of this manuscript. We have carefully considered all your comments and fully agree with the points raised. The manuscript has subsequently been revised to address each of them, copied below into corresponding sections with direct answers.   

**Reviewer 1, Comment 1**, Section - introduction: in the beginning of the manuscript there is quite a firm statement that there is a major role for the consumers to transform food systems by changing their diet. This comes across as little nuanced. It is known that consumers are subjected to the food system as it is now (capitalism driven), of which for example its massive advertisement (and low pricing strategy) for unhealthy food is highly influencing citizens. And, alternatively, citizens are not only consumers, they are citizens who can exercise different forms of power apart from consuming (think: producing, or activism, voting, etc.). I do see a note in the discussion (in 'actions and outputs toward food system transformation') on citizens being 'change agents'. I think it is wise to put this central in the introduction of the manuscript as well. Or be super self-critical on the project in case citizens were 'only' approached as consumers in the project. 

**Authors Response:** We agree but remain unsure exactly which statement the reviewer is referring to. As authors, we did not fully understand this comment or what it was specifically addressing, mainly because we completely agree with the reviewer’s point that food system transformation, by definition, involves systemic changes beyond the influence of any single stakeholder group. This means we fully agree that consumers are a part of the food system, and while they can influence their immediate behaviors, their actions alone cannot transform the entire system. Perhaps the original statement in the manuscript that the reviewer is referring to is: ‘Though the reduction in meat consumption appears to be at a tipping point (EC DG AGRI, 2021), associated impacts are still high (FAO, 2022; FAO, 2017).’ We recognize that this statement could be clearer, and we have rewritten it to avoid any potential misinterpretations. The revised text is as follows: 'Despite significant progress in reducing global hunger, millions of people still suffer from food insecurity and malnutrition (FAO et al., 2020). And despite progress being made on reducing consumption of high-carbon foods, the associated impacts remain high (FAO, 2017; EC DG AGRI, 2021; FAO, 2022). Moreover, Food Systems remain under stress in order to meet the demand of nutritious and affordable food of a global population (FAO, 2022). ‘

**Reviewer 1, Comment 2**, Section - introduction: on page 4 the authors write about the need to decentralise the food system. Due to colonialism in the last centuries, many global food production and distribution networks were created, and when European cities decide to cut these up to and go local instead, major inequalities may arise. I would expect certain reflection on global inequalities that arise in the transition towards decentralised food systems (and how this was touched upon in the project). Maybe the authors did this in the discussion, but then I overlooked it. 

**Authors Response: **Agree, In response to the reviewer’s comments, we have revised the manuscript thoroughly to ensure that the European scope is clear, and we have made a distinction when discussing global food systems, which will likely experience changes differently than European systems. To address this, we have ensured that all relevant mentions of food systems specify the geographical area being discussed, such as 'a transformation of European food systems' in the first paragraph. We have also added a new paragraph in the discussion addressing omissions beyond the project’s scope, which is included below: ‘Furthermore, the FoodSHIFT findings would benefit from a gap analysis that highlights areas that were either insufficiently detailed or excluded as beyond the project's scope. Most notably, the impact that transforming Europe's Food Systems would have on various parts of the global supply chain. Given the interconnected nature of global food supply chains, transformative actions in Europe would be best accompanied by equally transformative, well planned interventions conducted simultaneously across the entire food supply chain. This approach would maximize the benefits of sustainable food systems while minimizing potential negative effects, especially for producers in low-income, high-export countries.'

******Reviewer 1, Comment 3**, Section – introduction, discussion: I miss certain references to other work at certain points. Foremost in the discussion section. But also in the introduction more references could be made to research into living labs. I do a few alternative suggestions that I know of below.  1. Erisman J, Feenstra L, Broerse J, et al.: Labbing for sustainability transformations: Learning about challenges and strategies for impact. GAIA - Ecological Perspectives for Science and Society. 2024; 33 (1): 64-71 | Publisher Full Text 2. Kok K, van der Meij M, Wagner P, et al.: Exploring the practice of Labs for sustainable transformation: The challenge of ‘creating impact’. Journal of Cleaner Production. 2023; 388 | Publisher Full Text **Authors Response: **We agree with both reviewers who highlighted the importance of this point, and we have heavily revised this section as Living Labs are a critical part of the manuscript. This includes adding four new academic references and rewriting two existing references to clarify their connections. These revisions are 3 full paragraphs now form the final part of the introduction.

**Reviewer 1, Comment 4**, Section - methods: it is not fully clear to me why this letter is structured as a regular scientific publication (intro, method, results, conclusion, discussion), while it is not a scientific research. This chosen structure makes it hard for a reader to understand what this article is about at first glance, and this chosen structure is also resulting in a manuscript that contains many redundancies (some things are being written twice or three times). Especially the method section - which describes the method of the project, not the method of scientific research into the project - is confusing in this regard. I would expect in a method section more explanation about how you came to the results that you present. The method/process of the project itself is more a contextual background thing, which can be added in the introduction about the project. To avoid redundancies, I would recommend restructuring the manuscript, and abandon the structure of a regular scientific manuscript. **Authors Response: **We agree with the reviewer, and, acknowledge this was something we were unsure of how best to overcome ourselves. With this, we have reviewed the manuscript to avoid duplication and reworded titles so it is more clear what each section is about rather than using the scientific standard titles. The methods section receives a heavy markup + changing the title from 'methods' to 'Implementing the FoodSHIFT 2030 Project', similarly the results section moved to 'project outcomes'"

**Reviewer 1, Comment 5,** Section - throughout: with regard to the tables about the living labs in the manuscript, I would suggest putting the full names of the cities in there and not the abbreviations, or delete them in the tables and consider the caption as the best way to say which city/initiative each table describes (as you already do now). I understand that the project collaborators may have referred to one another in this way, but for outsiders ATH FSLL (for example) does not make much sense.  

**Authors Response: **Agree and completed, the full names for each lab were already included at the top of each table but we agree that the additional abbreviations are confusing and have been removed.

**Reviewer 1, Comment 6,** Section - discussion: the manuscript merely presents FoodSHIFT as a 'super success' story. However, we all know that EU projects are very complex, very chaotic in the beginning, etc. so this multiple re-emphasis on how successful the project was is not something an EU project experienced reader may believe. In that sense, the authors could be more humble, and also share the struggles that they had, the insecurities they felt, the tensions between researchers and living lab practitioners (for example), etc., to accomplish the project results.

**Authors Response: **We agree with the reviewer comments and while also include that the manuscript does contain a substantial part of the discussion which is meant as reflection and ways to improve and go beyond FoodSHIFT. We also agree that perhaps this section was not as clear as it should be and is now re-titled ' Experienced based reflections and key areas for improvements'  in combination with this, we also elaborate with new text on the omissions focusing in on how positive transformation in Europe may have negative impacts elsewhere along the global food supply chain. in addition the conclusion section is heavily edited to provide a shorter, more balanced view of the project and what it did / did not cover.
